# Social Connectivity in the Context of COVID-19 and Long-Term Care

**DOI:** 10.1093/geroni/igab046.1582

**Published:** 2021-12-17

**Authors:** Anna Garnett, Hannah Pollock, Natalie Floriancic, Lorie Donelle, Yolanda Babenko-Mould, Abe Oudshoorn, Carri Hand, Cheryl Forchuk

**Affiliations:** 1 University of Western Ontario, London, Ontario, Canada; 2 Western University, London, Ontario, Canada; 3 Western University, Western University, Ontario, Canada

## Abstract

The COVID-19 pandemic has disproportionately impacted older adults, particularly those residing in long-term care homes (LTCHs), causing immense loss of life and resulting in overall health declines in LTCH residents. These vulnerable older adults have also experienced extreme loneliness, anxiety and depression. Social connectedness is an important contributor to well-being and quality of life of older adults in LTCHs and family members are an essential component to this. However, restrictions driven by policies to protect resident safety, have constrained family members’ access to long-term care homes and limited in-person contact between residents and their families. In their absence, health providers have been integral to supporting connections between residents and their families within LTCHs. This study aimed to understand the experiences of social connectedness between residents and family members who have been physically separated due to the current pandemic and, to examine LTCH health providers’ experiences and responses to support social connectedness. Using a qualitative descriptive design, in-depth semi-structured interviews were conducted with 21 family members and 11 healthcare providers. Emergent themes from qualitative content analysis are: (a) all-encompassing impacts of separation; (b) advocacy became my life; (c) the emotional toll of the unknown; 4) the burden of information translation; 5) precarious balance between safety and mistrust for the healthcare system; and (d) a formulaic approach impedes connectivity. A more comprehensive understanding of the experiences and support needs of LTCH residents and their family members within the context of a pandemic can inform practice approaches to support social connections going forwards.

